# Supplementary material for: A Comparison of the Antioxidant Potential and Metabolite Analysis of Marine Fungi Associated with the Red Algae Pterocladiella capillacea from Northern Taiwan
Source: Antioxidants (Basel). 2024 Mar 11;13(3):336. doi: 10.3390/antiox13030336 (PMC10967630; doi:10.3390/antiox13030336)
Supplement: Supplementary file 1 [file antioxidants-13-00336-s001.zip › antioxidants-2871522-supplementary.pdf]

Supplementary file

Compounds	m\z	Retention Time (min)	Drift Time (ms)	Chromatographic Peak Width (min)	Max Fold Change	Maximum Abundance	Formula
Niazicin A	771.24	9.396	5.524	2.71665	2223.1664	155202.77	C34H46N2O14S2
Chembl4211493	601.29	21.31	5.0998725	0.4372667	2124.0947	97804.718	C33H40N6O3S
Sucrose octaacetate	701.19	25.75	4.9298768	1.0998667	3.9352786	109316.67	C28H38O19
Hemorphin-4	583.28	22.492033	4.9298768	0.5101167	921.92343	62948.708	C29H35N5O7
Phenyl-agarose	724.26	25.755217	5.2698683	1.0998667	5.4197024	16089.592	C30H42O19
(3b,20R,22R)-3,20,27-Trihydroxy-1-oxowitha-5,24-dienolide 3-glucoside	635.33	12.804933	4.6748831	0.77195	150.80576	12936.765	C34H50O11
Digitoxigenin bisdigitoxide	657.36	14.2031	4.8448789	0.54655	29.948839	12787.919	C35H54O10
LysoPS (16:0/0:0)	498.28269	42.26705	3.9949001	2.09045	52.387435	11497.575	C22H44NO9P
Sinalbine	735.20646	6.4607167	5.0998725	2.5709	130.44119	20336.591	C30H42N2O15S2
Olodaterol	387.19282	29.098083	2.1249469	0.6626333	6.3739395	14503.532	C21H26N2O5
Motolimod	481.26204	12.331283	2.2949426	0.4372333	14.972486	6348.0114	C28H34N4O2
Docosanamide	340.35655	45.785283	2.719932	3.081	10.509547	5797.607	C22H45NO
N-Nitrosofenfluramine	261.12365	11.7415	1.9549511	1.2456333	9.2114813	28770.256	C12H15F3N2O
Peonidin acetyl 3,5-diglucoside	685.21675	25.755217	4.759881	0.8448333	3.7295936	8467.5307	C30H35O17+
Ophiopogonin C'	745.41909	14.96825	6.2898428	0.4440333	27.962322	5341.087	C39H62O12
Aminopentol	406.35308	22.783517	3.1449214	0.9176833	7.3517769	17448.697	C22H47NO5
Ribociclib	452.29107	20.146483	3.0599235	0.4372667	1336.7866	4802.418	C23H30N8O
Suvorexant	451.16402	33.9713	2.8899278	1.0270167	19.589935	4624.7511	C23H23ClN6O2
Janthitrem G	630.37881	44.138867	5.6098598	1.027	11.102486	11179.177	C39H51NO6
Labadoside	755.25328	9.7603667	5.2698683	0.7355	119.09643	4414.334	C38H42O16
1-Sinapoyl-2,2′-diferuloylgentiobiose	918.30207	9.43245	7.5648109	1.79215	2092.6033	3672.4643	C43H48O21
Digitoxigenin 3-[glucosyl-(1->6)-glucosyl-(1->4)-2,6-dideoxyribohexoside]	851.39793	29.571733	7.6498088	0.9541333	9.1262573	11713.118	C41H64O17
Boviquinone 4	413.26408	33.534067	3.0599235	0.8812833	59.063042	3387.6572	C26H36O4

Table S1. LC-MS analysis and major compounds present in the fungal extracts.

Figure S1. Chemical structures of the abundant compounds identified in fungal extracts: Figure S1 is an extended version of Table 3, it shows the specific functional group information about the metabolites, complementing the overall idea conveyed by Table 3. The functional group information of specific metabolites can be easily identified from the figures provided.

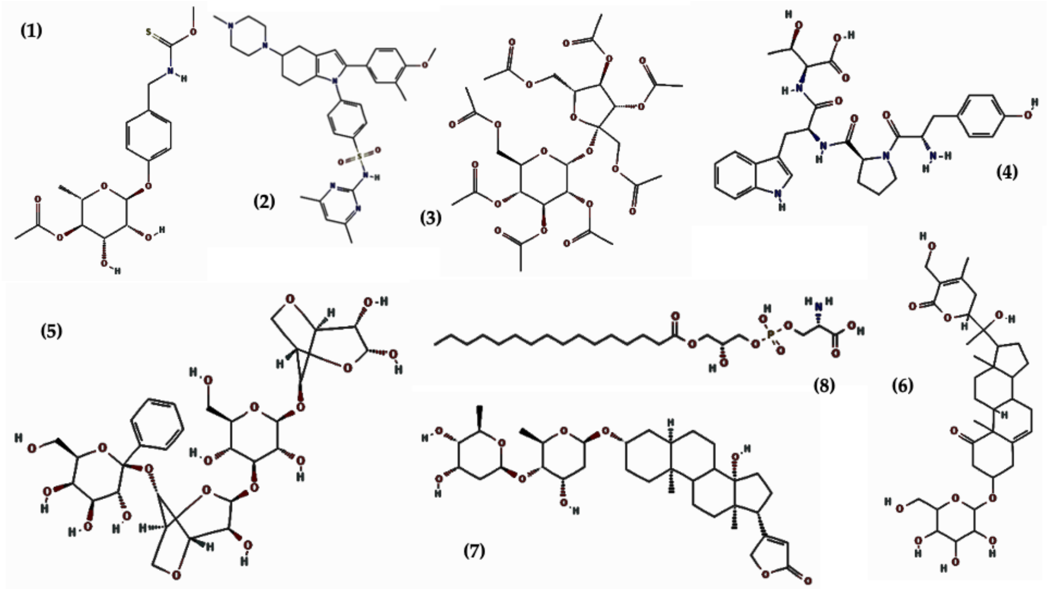

Figure S1. Chemical structures of the abundant compounds identified in fungal extracts: (1) niazicin A, (2) Chembl4211493, (3) sucrose octaacetate, (4) hemorphin-4, (5) phenyl-agarose, (6) (3b,20R,22R)-3,20,27-trihydroxy-1-oxowitha-5,24-dienolide 3-glucoside, (7) digitoxigenin bisdigitoxide, (8) lysoPS (16:0/0:0).

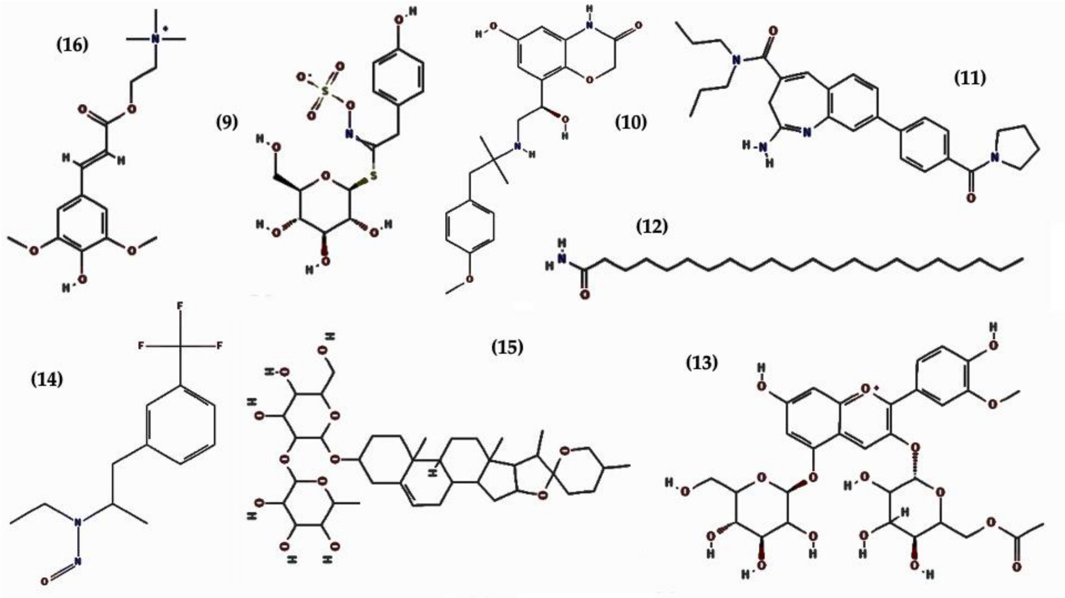

(9) and (16) sinalbine, (10) olodaterol, (11) motolimod, (12) docosanamide, (13) peonidin acetyl 3,5-diglucoside, (14) N-nitrosofenfluramine, (15) ophiopogonin C', .

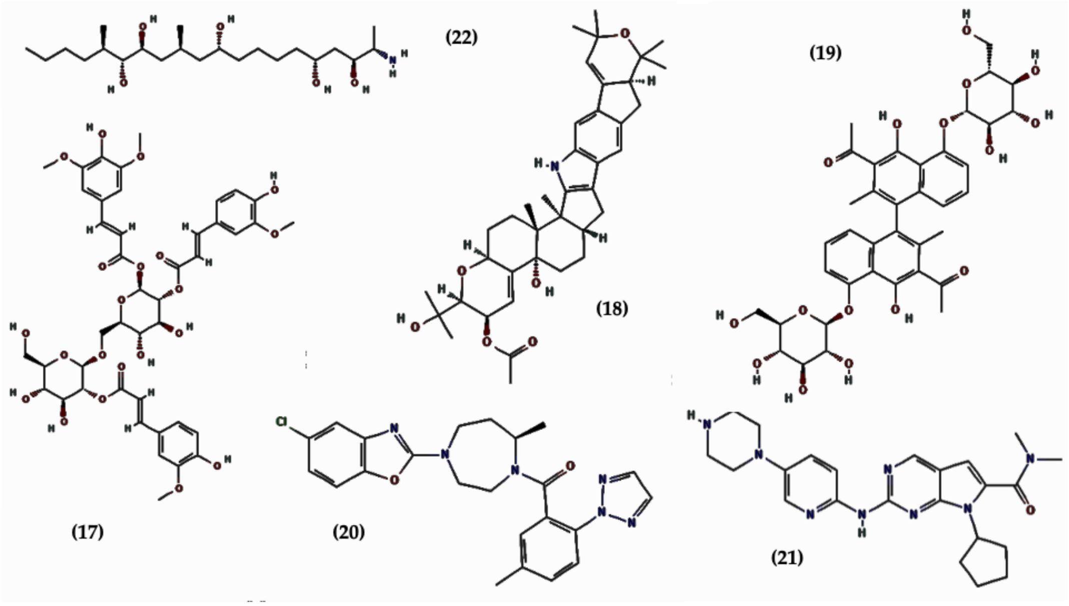

(17) 1-sinapoyl-2,2'-diferuloylgentiobiose, (18) janthitrem G, (19) labadoside, (20) su-vorexant, (21) ribociclib, and (22) aminopentol.
